# Alpha 1-Adrenoceptor Blocker May Improve Not Only Voiding But Also Storage Lower Urinary Tract Symptoms Caused by **^125^**I Brachytherapy for Prostate Cancer

**DOI:** 10.1155/2014/140654

**Published:** 2014-03-30

**Authors:** Nobuyuki Oyama, Yoshitaka Aoki, Hideaki Ito, Yoshiji Miwa, Hironobu Akino, Yoshitaka Sato, Hiroki Shioura, Hirohiko Kimura, Osamu Yokoyama

**Affiliations:** ^1^Department of Urology, Faculty of Medical Sciences, University of Fukui, 23-3 Matsuoka-Shimoaizuki, Fukui 910-1193, Japan; ^2^Department of Radiology, Faculty of Medical Sciences, University of Fukui, Fukui 910-1193, Japan

## Abstract

*Purpose*. To assess changes in lower urinary tract symptoms (LUTS) within 1 year after brachytherapy in patients receiving alpha 1-adrenoceptor antagonists. *Methods*. We retrospectively evaluated 116 patients who underwent ^125^I prostate brachytherapy in our institute. Seventy-one patients were treated with a combination of external beam radiation therapy and brachytherapy. Alpha 1-adrenoceptor antagonists were prescribed to all patients after brachytherapy. International Prostate Symptom Score (IPSS) forms and postvoid residual urine volume were recorded at all follow-up visits. *Results*. Forty-nine patients were given tamsulosin hydrochloride, 32 were given silodosin hydrochloride, and 35 were given naftopidil for up to 6 months after seed implantation. Patients given tamsulosin or naftopidil tended to show a higher peak IPSS and slower recovery to baseline values than those given silodosin. The patients given naftopidil showed an insufficient recovery in storage symptoms in naftopidil group in comparison with tamsulosin group at 3 months and with silodosin group at 6 and 9 months. *Conclusions*. In the management of LUT after brachytherapy, silodosin may provide a more favorable improvement. Silodosin and tamsulosin may have an advantage in improving not only voiding but also storage lower urinary tract symptoms after brachytherapy.

## 1. Introduction

Prostate cancer is the most commonly diagnosed cancer and the second leading cause of cancer death in men over the age of 40 years in the United States [1], and its incidence has been increasing in Japan. Since its approval in September 2003, interest in the use of ^125^I prostate brachytherapy for localized prostate cancer has increased, and the Japan-Prostate Cancer Outcome Study Group has estimated that more than 18,000 patients have received this therapy up to March 2011. This increase has been accompanied by growing recognition that lower urinary tract symptoms (LUTS) after seed implantation are a major complication of brachytherapy [2–6]. Management of this complication remains ineffective, however, substantially impacting quality of life after treatment.

Alpha 1-adrenoceptor antagonists are well known to provide relief from LUTS caused by benign prostate hyperplasia (BPH) and are recommended for patients complaining of LUTS after seed implantation [7–9]. *α*1-adrenoceptors are generally subdivided into alpha 1A-, alpha 1B-, and alpha 1D-adrenoceptor subtypes [10]. The alpha 1A-adrenoceptor subtype predominates in the prostatic stroma at the mRNA and protein level and is responsible for the dynamic component of obstruction and related voiding symptoms [11]. Recently, a number of experimental findings have indicated the involvement of the alpha 1D-adrenoceptor subtype in storage symptoms [12–14]. Alpha 1-adrenoceptor antagonists with significant affinity for the alpha 1D-adrenoceptor subtype are known to improve storage symptoms related to bladder outlet obstruction.

Silodosin was developed as a highly selective alpha 1A-adrenoceptor antagonist, with 55-fold greater affinity for alpha 1A- than alpha 1D-adrenoceptors [15]. Naftopidil has predominant affinity for alpha 1D-adrenoceptors [16]. Tamsulosin has relatively high affinity for alpha 1A- and alpha 1D-adrenoceptors compared to the alpha 1B-adrenoceptor [17] but exhibits only a relatively small 3.3-fold greater affinity for the alpha 1A- over the alpha 1D-adrenoceptor subtypes. In this study, we assessed the impact of three alpha 1-adrenoceptor antagonists with different levels of affinity for human alpha 1-adrenoceptor subtype in the management of LUTS after seed implantation. Furthermore, we investigated the role of the prostatic urethra in the development the storage dysfunction.

## 2. Methods and Materials

A total of 166 patients with localized prostate carcinoma underwent transperineal ^125^I prostate brachytherapy between May 2006 and December 2010 at our institute. Detailed information regarding urinary symptoms was collected before and after seed implantation using the International Prostate Symptom Score (IPSS). A total of 116 patients with IPSS scores at pretreatment and 1, 3, 6, 9, and 12 months after treatment were eligible. Mean age was 68.1 years (range 54–82). Among treatment-related variables, mean serum prostate-specific antigen (PSA) level at diagnosis was 10.7 ng/mL (range 3.0–88.9); mean Gleason sum was 6.9 (range 3–9); mean pretreatment prostate ultrasound volume was 28.1 cc (range 13.9–54.5 cc); and mean pretreatment IPSS was 9.4 (range 0–32). According to the risk classification of D'Amico, 24, 49, and 43 patients were classified as low-, intermediate-, and high-risk, respectively [7].

Patients were implanted with ^125^I seeds (OncoSeed; Nihon Medi-Physics Co., Japan) using preplanning and modified peripheral loading techniques using a Mick applicator. The mean number of implanted seeds was 70 (range 30–95). The prescribed doses were 144 Gy in patients undergoing seed implantation alone and 105 Gy in those undergoing combined external beam radiation therapy (EBRT). Combined EBRT was performed in 71 (61.2%) patients. Forty-five (38.8%) patients received neoadjuvant hormone therapy with luteinizing hormone-releasing hormone (LH-RH) agonist and antiandrogen before seed implantation for a mean of 9.9 months (median 6 months; range 3–50). No patients received hormone therapy after seed implantation.

From 1 day after seed implantation, tamsulosin hydrochloride was prescribed at a dose of 0.2 mg to 49 patients (tamsulosin group), silodosin hydrochloride at 8 mg to 32 patients (silodosin group), and naftopidil at 75 mg to 35 patients (naftopidil group), with all of these being the maximum permitted doses in Japan. Anticholinergic and anti-inflammatory agents were not used in any patients studied. Follow-up visits were made 1 and 3 months after seed implantation and at 3-month intervals thereafter. The International Prostate Symptom Score (IPSS) forms and postvoid residual urine volume were recorded at every visit.

All data are reported as means ± standard deviation (SD). Age, serum PSA value, Gleason sum, prostate volume, IPSS before treatment, and number of implanted seeds were compared among the three groups using the one-way analysis of variance (ANOVA). The statistical significance of intergroup difference of absolute IPSS between baseline and each time point after treatment was analyzed by the unpaired* t*-test. The statistical significance of increase of IPSS from baseline among three groups was analyzed by the one-way ANOVA followed by a Tukey's multiple comparison test. Statistical significance was established at *P* < 0.05. All statistical tests were performed using the SPSS package, version 12.0 (SPSS. Inc., Chicago, IL, USA).

## 3. Results

Tamsulosin, silodosin, and naftopidil were administered for up to 6 months after seed implantation. Patient characteristics, such as age, prostate volume, and number of seeds, did not significantly differ among the groups except for Gleason sum (tamsulosin versus naftopidil; 6.7 versus 7.3, *P* < 0.05) (Table 1).

**Figure 1:**
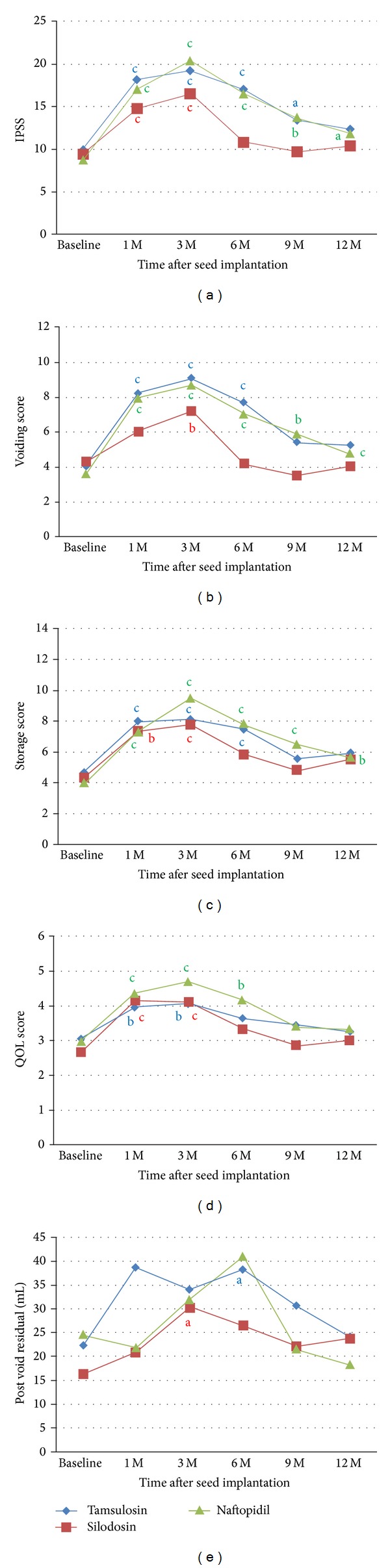
Absolute IPSS and postvoid residual urine volume at baseline and 1, 3, 6, 9, and 12 months after seed implantation in tamsulosin, silodosin, and naftopidil group. The mean values of total IPSS (a), voiding score (b), storage score (c), QOL score (d), and postvoid residual urine volume (e). The statistical significance of intergroup difference of each score or volume after treatment was analyzed by the unpaired* t*-test ((a) *P* < 0.05, (b) *P* < 0.01, and (c) *P* < 0.001).

IPSS values peaked at 3 months after seed implantation in all three groups (Figure 1(a)), at 19.2 ± 9.8 for the tamsulosin group, 16.5 ± 7.2 for the silodosin group, and 20.4 ± 8.8 for the naftopidil group and then returned to baseline at 1 year for the tamsulosin and naftopidil group and at 6 months for the silodosin group, respectively. Compared with pretreatment scores, a significant increase in IPSS was seen at 1, 3, 6, and 9 months in the tamsulosin group, 1, 3, 6, 9, and 12 months in the naftopidil group, and at 1 and 3 months only in the silodosin group. 

IPSS was also analyzed for voiding (intermittency, weak stream, and straining) and storage (frequency, urgency, and nocturia) scores separately. Voiding score peaked at 3 months after seed implantation in all three groups (Figure 1(b)). Voiding score peaked at 9.1 ± 5.0 for the tamsulosin group, 8.7 ± 4.8 for the naftopidil group, and 7.2 ± 4.1 for the silodosin group and then returned to baseline values at 12 for the tamsulosin and naftopidil groups and at 6 months for silodosin group, respectively. Compared with pretreatment scores, a significant increase in voiding score was seen at 1, 3, and 6 months in the tamsulosin group, at 1, 3, 6, 9, and 12 months in the naftopidil group, and at 3 months only in the silodosin group.

Storage score peaked at 8.1 ± 4.1 for the tamsulosin group, 9.5 ± 3.8 for the naftopidil group, and 7.8 ± 3.7 for the silodosin group and then returned to baseline values at 9 months for the tamsulosin and silodosin groups, while storage score remained increased up to 12 months for naftopidil group, respectively (Figure 1(c)). Compared with pretreatment scores, a significant increase in storage score was seen at 1, 3, and 6 months in the tamsulosin group, at 1, 3, 6, 9, and 12 months in the naftopidil group, and at 1 and 3 months in the silodosin group.

QOL score peaked at 3 months in tamsulosin (4.1 ± 1.5) and naftopidil groups (4.7 ± 1.9) and at 1 month in silodosin group (4.2 ± 1.3) (Figure 1(d)) and then returned to baseline at 1 year for all three groups, respectively. Compared with pretreatment scores, a significant increase in QOL score was seen at 1 and 3 months in the tamsulosin and silodosin groups and 1, 3, and 6 months in the naftopidil group.

Postvoid residual urine volume peaked at 1 month in tamsulosin (38.8 ± 9.5 mL) and at 6 months in naftopidil groups, (41.1 ± 9.5 mL) and at 3 months in silodosin group (30.3 ± 5.2 mL) (Figure 1(e)) and then returned to baseline at 1 year for tamsulosin and naftopidil groups, respectively. Compared with pretreatment scores, a significant increase in postvoid residual urine volume was seen at 6 months in the tamsulosin group and 3 months in silodosin group.

**Figure 2:**
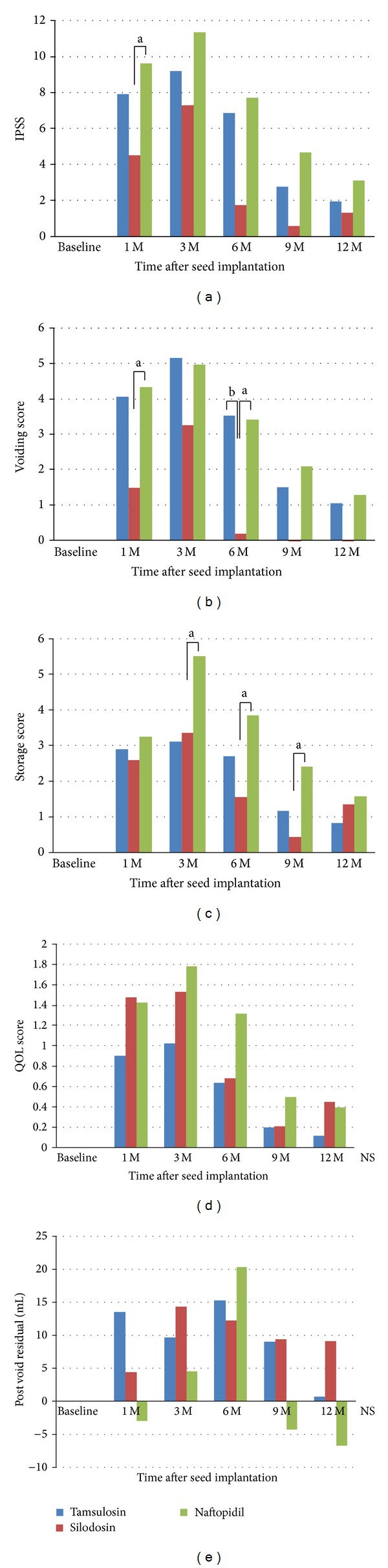
Increase of IPSS and postvoid residual urine volume from baseline at baseline and 1, 3, 6, 9, and 12 months after seed implantation in tamsulosin, silodosin, and naftopidil group. The mean values of increase of total IPSS (a), voiding score (b), storage score (c), QOL score (d), and postvoid residual urine volume (e). The statistical significance of increase of IPSS from baseline among three groups was analyzed by the one-way ANOVA followed by a Tukey's multiple comparison test ((a) *P* < 0.05, (b) *P* < 0.01, and (c) *P* < 0.001).

Changes in LUTS in the first year after seed implantation were compared using IPSS values and postvoid residual urine volume that varied from baseline. The increase in IPSS at 1 month in the naftopidil group was higher than that in silodosin group (naftopidil versus silodosin; 9.6 versus 4.5, *P* < 0.05) (Figure 2(a)). When the voiding and storage scores were analyzed separately, the increase in voiding score in the naftopidil or tamsulosin group was higher than that in the silodosin group at 1 and 6 months (naftopidil or tamsulosin versus silodosin; 4.3 or 4.0 versus 1.5, *P* < 0.05 or *P* = 0.05, at 1 month and 3.4 or 3.5 versus 0.2, *P* < 0.05 or *P* < 0.01, at 6 months) (Figure 2(b)). Besides, the increase in storage score in the naftopidil group was higher than that in the tamsulosin group at 3 months (naftopidil versus tamsulosin; 5.5 versus 3.1, *P* < 0.05) (Figure 2(c)). The increase in storage score in the naftopidil group was also higher than that in the silodosin group at 6 and 9 months (naftopidil versus silodosin; 3.9 versus 1.6, *P* < 0.05, at 6 months, and 2.4 versus 0.4, *P* < 0.05, at 9 months). There were no significant differences in QOL score or postvoid residual urine volume among three groups (Figures 2(d) and 2(e)).

## 4. Discussion

LUTS after seed implantation, which is characterized by the combination of both voiding and storage symptoms [5], are one of the most bothersome complications of prostate brachytherapy. The presumed causes are the traumatic effect of needle insertion and seed implantation and inflammatory changes in the urethra and prostate following radiation exposure [3]. The present study demonstrated the efficacy of alpha 1-adrenoceptor antagonists in the treatment of acute LUTS following brachytherapy. Among findings, the worsening of IPSS after seed implantation tended to be stronger in patients receiving tamsulosin and naftopidil than in those receiving silodosin and to last longer, up to 1 year before returning to baseline level compared with 6 months in the silodosin group. These findings suggest that silodosin may have an advantage in the management of LUTS after ^125^I prostate brachytherapy in comparison to tamsulosin or naftopidil.

Alpha 1-adrenoceptor antagonists are well known for their symptomatic efficacy against LUTS caused by BPH, and a wide variety of agents are now available. The potential of these agents in relieving LUTS after seed implantation has recently been reported [8]. In their study of alpha 1-adrenoceptor antagonists for LUTS in 170 patients undergoing brachytherapy for prostate cancer, Merrick et al. reported that IPSS returned to baseline levels within a median of 6 weeks and a mean of 13.3 weeks and that 50% of patients returned to baseline values. Our study further supports existing findings of the value of alpha 1-adrenoceptor antagonists for LUTS after seed implantation [7–9, 18–20].

Although alpha 1-adrenoceptors antagonists show efficacy in alleviating acute LUTS after seed implantation, the effect of their characteristic selectivity for the alpha 1A-, alpha 1B-, and alpha 1D-adrenoceptors remains unknown. In our study, IPSS after seed implantation tended to worsen more strongly in patients receiving tamsulosin and naftopidil than in those receiving silodosin. Further, the high voiding score lasted for up to 1 year in the tamsulosin and naftopidil groups but returned to baseline at 6 months in the silodosin group. This difference might be explained by the difference in the selectivity of these agents for alpha 1-adrenoceptor subtypes. Three subtypes of adrenoceptors have been pharmacologically differentiated, alpha 1A, alpha 1B, and alpha D, of which alpha 1A and 1D predominate in prostatic tissues and the urinary bladder wall while 1B predominates in the blood vessel walls [21]. Tamsulosin has relatively high affinity for alpha 1A- and alpha 1D-adrenoceptors compared to the alpha 1B-adrenoceptor [17] but exhibits only a relatively small 3.3-fold greater affinity for the alpha 1A- over the alpha 1D-adrenoceptor subtypes. Naftopidil also has relatively high affinity for alpha 1A- and alpha 1D-adrenoceptors compared to the alpha 1B-adrenoceptor [17] but exhibits relatively high affinity for alpha 1D- than alpha 1A-adrenoceptors, which are mainly expressed in prostatic tissues. In contrast, silodosin was developed as a highly selective alpha 1A-adrenoceptor antagonist, with 55-fold greater affinity for alpha 1A- than alpha 1D-adrenoceptors. Shibata et al. reported that silodosin displayed a 583-fold greater affinity for the human alpha 1A-adrenoceptor than the alpha 1B-adrenoceptor [15]. This high selectivity is considered to be contributed to the high efficacy of this agent in the management of voiding symptoms. Moreover, storage score after seed implantation tended to worsen more strongly in patients receiving naftopidil than in those receiving tamsulosin or silodosin. According to the recent study, it has been reported that naftopidil improves storage symptoms as well as voiding symptoms for patients with overactive bladder in randomized prospective study [22]. This relatively lower performance of naftopidil in relieving storage symptoms after brachytherapy can be explained by the difference in the cause of storage symptoms between overactive bladder and postbrachytherapy patients. The storage symptoms related to brachytherapy are mainly associated with the traumatic effect and radiation-induced inflammation in the prostate as already mentioned. Smooth muscle tone in the prostate is mainly regulated by the alpha 1A-adrenoceptor [23, 24]. Therefore, the effect of naftopidil, which has relatively low affinity for alpha 1A-adrenoceptors, for storage symptoms after brachytherapy is presumed to be limited.

In the recent paper, to compare the efficacy of tamsulosin, silodosin, and naftopidil in treating LUTS after brachytherapy, a randomized controlled trial has been reported [25]. In the study, 212 patients of prostate cancer after brachytherapy received one of three alpha 1-adrenoceptor antagonists for one year. They found significant They found there were significantly greater decreases with silodosin than naftopidil at 1 month in the total IPSS. Silodosin showed a significant improvement in the postvoid residual at 6 months vs. tamsulosin. Silodosin showed a significant improvement in the IPSS voiding score at 1 month vs. naftopidil. No significant difference was observed in the storage score at any time points among the three groups. They concluded that silodosin has a greater impact on improving LUTS after brachytherapy than tamsulosin or naftopidil. The results of our current study coincide with those previously reported. For instance, both of the studies confirmed a superiority of silodosin to naftopidil in improving IPSS and voiding score at 1 month. Besides, the increase in voiding score in the naftopidil or tamsulosin group was higher than that in the silodosin group at 6 months. This suggests that silodosin might be more suitable than tamsulosin or naftopidil in managing voiding symptoms even at 6 months after brachytherapy. Our additional finding is an insufficient recovery in storage symptoms in naftopidil group in comparison with tamsulosin group at 3 months and with silodosin group at 6 and 9 months after brachytherapy. Previous reports did not mentioned therapeutic effect of alpha 1-adrenoceptor antagonists in the treatment of storage symptoms after seed implantation; their study also demonstrated relatively better improving in storage symptoms in silodosin group than tamsulosin or naftopidil group without statistical significance. Further study is needed to elucidate the role of alpha 1-adrenoceptor antagonists in the treatment of storage symptoms.

There are several limitations to our study. First, sample size was relatively small, and patients' number differed between groups. Second, treatment was not uniform, including the ratio of patients receiving neoadjuvant hormone therapy, the ratio of accompanying EBRT, and the number of seeds. Confirmation of the effectiveness of these alpha 1-adrenoceptor antagonists in the treatment of acute LUTS after seed implantation will thus require a randomized control trial with a large number of patients.

In conclusion, these results show that silodosin may provide a more favorable improvement in the management of LUTS after brachytherapy for prostate cancer. In addition, silodosin and tamsulosin may have an advantage in improving not only voiding but also storage lower urinary tract symptoms after brachytherapy.

## Figures and Tables

**Table 1:** Patient characteristics.

Characteristics	Tamsulosin (*n* = 49)	Silodosin (*n* = 32)	Naftopidil (*n* = 35)	*P* value
Age (y)				
Mean	68.9	66.4	68.7	NS
Range	58–82	54–80	54–77	
PSA at biopsy (ng/mL)				
Mean	12.0	11.3	8.2	NS
Range	3.1–61.9	3.1–88.9	3.0–36.4	
Gleason sum				
Mean	6.7*	7	7.3*	0.05*
Range	3–9	6–9	6–9	
Prostate volume at BT (cc)				
Mean	28.4	27.4	31	NS
Range	13.9–54.5	14.2–46.1	18.2–48.6	
IPSS before treatment				
Mean	9.9	9.4	8.8	NS
Range	1–32	0–29	0–25	
Neoadjuvant HT				
Yes	22	9	14	
No	27	23	21	
Number of seeds				
Mean	67.6	69.4	72.9	NS
Range	30–95	45–95	55–95	
EBRT				
Yes	26	23	22	
No	23	9	13	
Risk category				
Low risk	15	7	2	
Intermediate risk	14	15	20	
High risk	20	10	13	

PSA: prostatic specific antigen; BT: brachytherapy; IPSS: International Prostate Symptoms Score; HT: hor-mone therapy; EBRT: external beam radiation threrapy.

*Tamsulosin versus naftopidil; 6.7 versus 7.3, *P* < 0.05.
